# Interplay between C1-inhibitor and group IIA secreted phospholipase A_2_ impairs their respective function

**DOI:** 10.1007/s12026-022-09331-7

**Published:** 2022-11-17

**Authors:** Anne Lise Ferrara, Maria Bova, Angelica Petraroli, Daniela Marasco, Christine Payré, Sara Fortuna, Francesco Palestra, Renato Ciardi, Gianni Marone, Giuseppe Spadaro, Gérard Lambeau, Stefania Loffredo

**Affiliations:** 1 Department of Translational Medical Sciences and Center for Basic and Clinical Immunology Research (CISI), WAO Center of Excellence, University of Naples Federico II, Via S. Pansini 5, 80131 Naples, Italy; 2 CNR Institute of Experimental Endocrinology and Oncology “G. Salvatore”, Naples, Italy; 3 Department of Internal Medicine, Cardarelli Hospital, Naples, Italy; 4 Department of Pharmacy, University of Naples Federico II, Naples, Italy; 5 Institut de Pharmacologie Moléculaire Et Cellulaire, CNRS, Université Côte d’Azur, Valbonne Sophia Antipolis, Nice, France; 6 Istituto Italiano Di Tecnologia (IIT), Via E. Melen, 83, 16152 Genoa, Italy

**Keywords:** Phospholipase A_2_, C1-INH, Angioedema, Cytokines, Chemokines, Blood mononuclear cells

## Abstract

High levels of human group IIA secreted phospholipase A_2_ (hGIIA) have been associated with various inflammatory disease conditions. We have recently shown that hGIIA activity and concentration are increased in the plasma of patients with hereditary angioedema due to C1-inhibitor deficiency (C1-INH-HAE) and negatively correlate with C1-INH plasma activity. In this study, we analyzed whether the presence of both hGIIA and C1-INH impairs their respective function on immune cells. hGIIA, but not recombinant and plasma-derived C1-INH, stimulates the production of IL-6, CXCL8, and TNF-α from peripheral blood mononuclear cells (PBMCs). PBMC activation mediated by hGIIA is blocked by RO032107A, a specific hGIIA inhibitor. Interestingly, C1-INH inhibits the hGIIA-induced production of IL-6, TNF-α, and CXCL8, while it does not affect hGIIA enzymatic activity. On the other hand, hGIIA reduces the capacity of C1-INH at inhibiting C1-esterase activity. Spectroscopic and molecular docking studies suggest a possible interaction between hGIIA and C1-INH but further experiments are needed to confirm this hypothesis. Together, these results provide evidence for a new interplay between hGIIA and C1-INH, which may be important in the pathophysiology of hereditary angioedema.

## Introduction

The superfamily of phospholipases A_2_ (PLA_2_s) comprises intracellular and secreted enzymes that hydrolyze the *sn*-2 ester linkage of glycerophospholipids, releasing free fatty acids and lysophospholipids. Among multiple functions, PLA_2_s induce the release of arachidonic acid and other polyunsaturated fatty acids, which are precursors of potent pro- and anti-inflammatory lipid mediators such as the multiple subfamilies of eicosanoids, including most prominently the prostaglandins, thromboxanes, leukotrienes, lipoxins, resolvins, and eoxins [1–4]. The family of secreted phospholipases A_2_ (sPLA_2_s) comprises 12 members: IB, IIA, IIC, IID, IIE, IIF, III, V, X, XIIA, XIIB, and otoconin-95. Most of these extracellular enzymes have a low molecular mass (14–20 kDa); are structurally homologous, disulfide-rich, and Ca^2+^-dependent; and exhibit unique tissue and cellular distributions, substrate selectivity, and biological roles [1–4].

Human sPLA_2_s play key roles in pathophysiological processes. Indeed, they activate several subsets of immune cells [5–8] and are expressed in inflamed tissues and tumors [1–4, 9–12]. Human group IIA sPLA_2_ (hGIIA) is the most studied enzyme [13, 14]. It is detected in the peripheral blood, particularly under inflammatory conditions like sepsis, cardiovascular diseases, and malignancies [9, 15–19].

We recently showed that hGIIA sPLA_2_ activity and plasma levels are increased in patients with C1-esterase inhibitor deficiency hereditary angioedema (C1-INH-HAE) during symptom-free period compared to healthy controls [20]. sPLA_2_ activity was found to negatively correlate with C1-INH protein level and function. C1-INH is encoded by the *SERPING1* gene, which is located on chromosome 11q12.1 [21]. C1-INH is a 105-kDa glycoprotein and the main inhibitor of the classical complement enzymes C1r and C1s and of contact factors such as aFXIIa, bFXIIa, kallikrein, FXIa, plasmin, MASP-1, and MASP-2 [22–24].

On the basis of the combined functions of C1-INH, the congenital deficiency of C1-INH (incidence of 1:50,000) results in a kallikrein-kinin (contact) system–related disorder but with no clear signs of hemostatic problem. This disease is called C1-INH-HAE (OMIM #106,100) and is characterized by unpredictable recurrent spontaneous histamine-independent episodes involving the deeper layers of the skin and/or submucosal tissue that can take place at various tissue sites throughout the body [25].

Besides the above finding associating hGIIA and C1-INH, hGIIA sPLA_2_ and its mammalian orthologs have been shown to bind and modulate activities of different molecules involved in the complement or coagulation systems. For instance, the major sPLA_2_ (likely rat GIIA) purified from rat peritoneal inflammatory sites was inhibited by two large fragments of C3 complement factor [26]. hGIIA was also reported to exert anticoagulant effects by inhibiting prothrombinase activity via binding to FXa [27]. The effect was independent of phospholipid hydrolysis and due to direct interaction with FXa, as measured under both in vitro and ex vivo conditions [27]. On the other hand, regulation of C1-INH activity by other types of enzymes has already been shown. C1-INH can be degraded by serine proteases such as elastase and plasmin [28, 29] or can interact with them, for example, with MBL-associated serine protease 1 (MASP-1) and MASP-2, forming protein complexes [30].

Based on the above findings, we sought to determine whether hGIIA can interact and interfere with the function of C1-INH, and vice versa, either directly or indirectly, thereby leading to impaired activation of immune cells by either of the two types of molecules.

## Materials and methods

### Reagents

The following reagents were purchased: l-glutamine, antibiotic–antimycotic solution (10,000 IU/mL penicillin, 10 mg/mL streptomycin, and 25 µg/mL amphotericin B), detoxified LPS (from *E. coli* serotype 0111:B4), polymyxin B sulfate (Sigma-Aldrich, Milan, Italy), RPMI and fetal calf serum (FCS, endotoxin level < 0.1EU/mL, MP Biomedicals Europe, Illkirch, France), recombinant human C1-esterase inhibitor (rhC1-INH, PeproTech, USA), human plasma–derived C1-esterase inhibitor (pdC1-INH; Cinryze®, Takeda Pharmaceutical Company, Tokyo, Japan). All other reagents were from Carlo Erba (Milan, Italy).

Recombinant human group IIA sPLA_2_ (hGIIA) was produced in *E. coli* as the N1A catalytically active mutant (the N1A mutation facilitates the removal of the initiator methionine without impacting enzymatic activity) as reported [31]. RO032107A, a specific hGIIA inhibitor, was a kind gift from Pr. Michael Gelb (University of Washington, Seattle, USA) [32].

### Isolation and purification of peripheral blood mononuclear cells (PBMCs)

The study protocol involving the use of human blood cells was approved by the Ethics Committee of the University of Naples Federico II, and written informed consent was obtained from blood donors according to the principles expressed in the Declaration of Helsinki (Protocol Number 301/12). PBMCs were isolated from buffy coats of healthy donors (HBsAg^−^, HCV^−^, and HIV^−^) obtained from a leukapheresis unit. Plasma was separated from cellular components by centrifugation (400 × g for 20 min at 22 °C), collected, and stored at − 80 °C. Leukocytes were separated from erythrocytes by dextran sedimentation. PBMCs were purified by Histopaque-1077 (Sigma-Aldrich, Milan, Italy) density gradient centrifugation (400 × g for 20 min at 22 °C). The cells were resuspended (10^6^ cells/250 µL) in RPMI 1640 with 2 mM l-glutamine and 1% antibiotic–antimycotic solution, and incubated (37 °C, 5% CO_2_) in 48-well plates. After 2 h, the cell medium was removed and the plates were gently washed with fresh medium. The adherent cells were resuspended in RPMI 1640 with 5% FCS, 2 mM l-glutamine, and 1% antibiotic–antimycotic solution (complete medium) and used for experiments.

### Cell treatments

Resuspended PBMCs (1.5 × 10^6^ cells) were incubated for 16 h at 37 °C in complete medium with LPS (100 ng/mL), hGIIA (0.5, 1, 3 µg/mL), and rhC1-INH or pdC1-INH (250–500 µg/mL). In selected experiments, PBMCs were incubated for 16 h at 37 °C with rhC1-INH (250–500 µg/mL), pdC1-INH (250–500 µg/ml) in combination with hGIIA (3 µg/mL), and LPS (100 ng/mL). At the end of the experiments, cells were centrifuged (300 g, 4 °C, 5 min), and the supernatants were harvested and stored at − 80 °C for subsequent analyses. In selected experiments, hGIIA and LPS were preincubated (37 °C, 30 min) with either RO032107A (hGIIA inhibitor) (100 nM) or polymyxin B sulfate (50 µg/mL) before addition to cells.

Viability of cells treated with hGIIA, LPS, rhC1-INH, pdC1-INH, or their combination was evaluated by measuring mitochondrial activity using the MTT (3-(4,5-dimethylthiazol-2-yl)2,5-diphenyl tetrazolium bromide) assay, as reported [33]. PBMCs were incubated (37 °C, 16 h) with hGIIA, LPS, rhC1-INH, pdC1-INH, or their combination. At the end of incubation, supernatants were removed and the cells were incubated (37 °C, 1 h) in 1 mL of MTT solution (0.5 mg/mL). The cells were washed with PBS, 0.5 mL of DMSO was added, and absorbance was read at 540 nm. Cell injury is expressed as a percentage of sham-treated cultures (data not shown).

### ELISA assays

Concentrations of cytokines and chemokines in cell supernatants were measured using commercially available ELISA kits for IL-6 (range of detection 9.4–600 pg/mL), TNF-α (15.6–1000 pg/mL), and CXCL8 (31.3–2000 pg/mL) (R&D Systems, MN, USA). The results obtained were normalized for the total protein content in each well, determined in cell lysates (cells lysed with 0.1% Triton X-100) by a Bradford assay, with the standard curve performed with bovine serum albumin. Cytokine release was expressed as pg or ng of cytokine/mg of total proteins.

### Effect of C1-INH on hGIIA enzymatic activity

hGIIA enzymatic activity was measured as previously described, using [^3^H]-oleate-radiolabeled *E. coli* membranes as a sensitive substrate for sPLA_2_s [34]. To test the inhibitory effect of C1-INH on hGIIA sPLA_2_, the recombinant enzyme (hGIIA N1A, 10 pM) was preincubated with various concentrations of recombinant or plasma-derived C1-INH in 100 µL of sPLA_2_ activity buffer (100 mM Tris pH 8.0, 10 mM CaCl_2_, and 0.1% bovine serum albumin (BSA)) for 15 min at room temperature. The enzymatic activity was measured by addition of 30,000 dpm of [^3^H]-oleate-radiolabeled *E. coli* membranes in 100 µL of sPLA_2_ activity buffer and further incubation at 37 °C for 60 min. Reactions (200 µL) were stopped by addition of 300 µL of stop buffer (100 mM EDTA pH 8.0 and 0.1% fatty acid–free BSA). Mixtures were centrifuged at 10,000 × g for 5 min, and supernatants containing released free [^3^H]-oleate were counted (dpm/assay). The percentage of inhibition by C1-INH is calculated relative to the enzymatic activity measured in the presence of hGIIA but absence of C1-INH, after subtraction of the background value measured in the absence of sPLA_2_. Addition of C1-INH alone has no effect on the background value measured in the absence of hGIIA.

### C1-INH functional assays

C1-INH function was determined as the capacity of plasma C1-INH from healthy donors to inhibit the esterase activity of exogenous C1s with a chromogenic substrate (commercially available kit from Technoclone GmbH, Vienna, Austria). Reference ranges were as follows: 0.70 to 1.30 unit of C1-INH/mL (1 C1-INH unit corresponds to the average C1-INH activity present in 1 mL of fresh citrated normal plasma). The functional activity of plasma C1-INH was also expressed as a percentage of activity of C1-INH present in samples. In selected experiments, plasma of healthy donors was incubated (2 h, 37 °C) with and without hGIIA (3 μg/mL) or LPS (100 ng/mL). After treatment, the enzymatic activity of C1-INH was determined as above. In other experiments, hGIIA was preincubated for 2 h at 37 °C with rhC1-INH and pdC1-INH (or their absence) followed by determination of C1-INH activity. In a last group of experiments, hGIIA was preincubated with RO032107A and then incubated for 2 h at 37 °C with rhC1-INH and pdC1-INH (or their absence) followed by determination of C1-INH activity.

### Surface plasmon resonance (SPR)

Real-time binding assays were performed on a Biacore 3000 Surface Plasmon Resonance (SPR) instrument (GE Healthcare, Milan, Italy). The N1A mutant of hGIIA was immobilized at 800 RU on a CM5 Biacore sensor chip, at ~ 20 µg/mL in 10 mM sodium acetate, pH 5.0, by using the EDC/NHS chemistry, with a flow rate of 2 μL/min and an injection time of 7 min. BSA was immobilized similarly as a reference channel. Binding assays were carried out by injecting 90 µL of analyte, at 30 µL/min, with various concentrations of C1-INH 10, 20, 30, 40, and 50 µM in HBS (10 mM Hepes, pH 7.4, 150 mM NaCl, 3 mM EDTA). The association rate (*k*_on_) was monitored for 180 s, and the dissociation rate (*k*_off_) was monitored for 300 s. The BIAevaluation analysis package (version 4.1, GE Healthcare) was used to subtract the signal from the reference channel and kinetic values were estimated by applying a 1:1 Langmuir model, as reported [35].

### Fluorescence spectroscopy

A Jasco FP 8300 spectrofluorometer equipped with a 10-mm path-length quartz cuvette was employed. Data were acquired at 25 °C, using an excitation wavelength of 298 nm and a fluorescence emission wavelength ranging from 300 to 400 nm, at a 2 μM concentration of C1-INH, in 10 mM phosphate buffer, pH 7.4, and incubated in the presence of increasing concentrations of hGIIA (0–20 µM) ranging from 0 to 1.0 equivalents. Experiments were carried out in duplicates.

### Molecular modeling

For hGIIA (UniProt P14555), we employed chain A (aa 1–124) of PDB entry 1DCY [36]. For plasma C1-INH (UniProt P05155), we employed chain A (aa 100–477) of PDB entry 2OAY [37]. For both proteins, the interacting surface was predicted with cons-PPISP [38], SPIDER [39], and PINUP [40] by integrating them with CPORT through a web-based interface [41]. The protein interfaces determined by CPORT were then employed to dock the two proteins through the HADDOCK web-based interface [42, 43]. 2D ligand–protein interaction diagrams were generated with LigPlot + [44].

### Molecular dynamics

The C1-INH-hGIIA model with the lowest HADDOCK score was placed in a cubic box with a water layer of 1.0 nm, neutralized with Na^+^ and/or Cl^−^ ions, and minimized. The steepest descent minimization stopped either when the maximum force was lower than 1000 kJ/mol/nm or when 50,000 minimization steps were performed with 0.005 kJ/mol energy step size, Verlet cut-off scheme, short-range electrostatic cut-off, and van der Waals cut-off of 1.0 nm. AMBER99SB-ILDN force field [45], tip3p water, and periodic boundary conditions were employed. NVT and NPT equilibrations were performed for 100 ps by restraining the protein backbone, followed by 500-ns-long NPT production runs at 330 K. The iteration time step was set to 2 fs with the Verlet integrator and LINCS [46] constraint. All the simulations and their analyses were run as implemented in the Gromacs package 2020.3 [47]. Root mean squared deviations (RMSDs) were calculated from configurations sampled every 0.5 ns. Simulations were run on M100 (CINECA, Italy).

### Statistical analysis

The data are expressed as mean values ± SEM (standard error mean) of the indicated number of experiments. Statistical analysis was performed with Prism 6 (GraphPad Software). Statistical analysis was performed by Student’s *t*-test or one-way analysis of variance followed by Dunnett’s test (when comparison was made against a control) or Bonferroni’s test (when comparison was made between each pair of groups). Statistically significant differences were accepted when the *p*-value was at least ≤ 0.05.

## Results

### Effects of hGIIA on the release of cytokines and chemokines from PBMCs

**Fig. 1:**
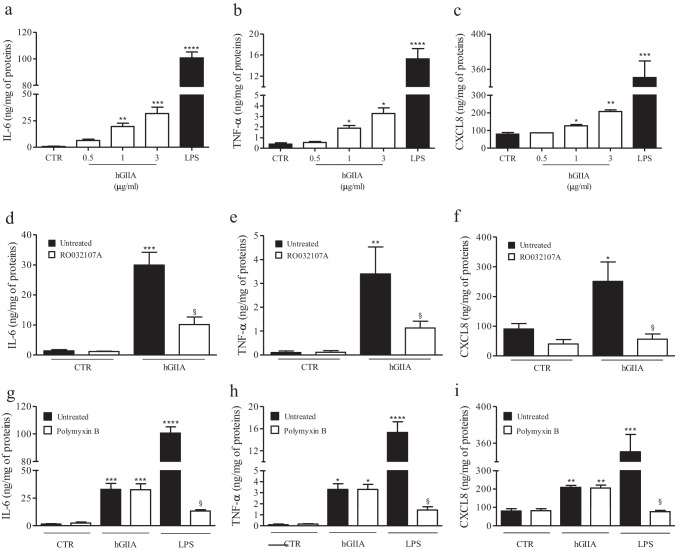
**a**–**c** Effects of hGIIA and LPS on secretion of IL-6, TNF-α, and CXCL8 from human PBMCs. PBMCs were stimulated (16 h, 37 °C) with or without the indicated concentrations of hGIIA (3 µg/mL) or LPS (100 ng/mL). **d**–**f** Effects of RO032107A on PBMCs activated by hGIIA. hGIIA (3 µg/mL) was preincubated with or without RO032107A (100 nM) before addition to PBMCs. **g**–**i** Effects of polymyxin B on PBMCs activated by hGIIA and LPS. hGIIA (3 µg/mL) and LPS (100 ng/mL) were preincubated (37 °C, 30 min) with polymyxin B sulfate (50 mg/mL) before addition to cells. IL-6 (**a**, **d**, **g**), TNF-α (**b**, **e**, **h**), and CXCL8 (**c**, **f**, **i**) concentrations in cell medium were determined by ELISA and values were normalized relative to the amount of total proteins in each well (measured by Bradford assay on cell lysates). The data are reported as mean ± SEM of six different preparations of PBMCs from six different donors. *p < 0.05 vs. **p < 0.01, ***p < 0.001, ****p < 0.0001 vs. control

Upon activation, PBMCs release cytokines and chemokines such as IL-6, TNF-α, and CXCL8 [48]. In a first series of experiments, we evaluated the effects of hGIIA on the secretion of cytokines and chemokines from PBMCs. hGIIA induced the release of IL-6 (Fig. 1a), TNF-α (Fig. 1b), and CXCL8 (Fig. 1c). For comparison, LPS, the most abundant component within the cell wall of Gram-negative bacteria and immune cell activator, was used as positive control and potently released the same cytokines [48, 49]. The effect of hGIIA on the release of cytokines and chemokines was sensitive to the active site sPLA_2_ inhibitor RO032107A [50] (Fig. 1d–f). Moreover, although we used highly purified recombinant hGIIA in these experiments, we excluded the possibility that the effect of hGIIA was due to small amount of LPS contamination by stimulating PBMCs with hGIIA in the presence of polymyxin B (50 µg/mL), a potent binder of LPS [51]. Polymyxin B did not influence the capacity of hGIIA to induce the release of IL-6 (Fig. 1g), TNF-α (Fig. 1h), and CXCL8 (Fig. 1i), whereas it almost completely suppressed the production of cytokines and chemokines induced by LPS (Fig. 1g–i).

### Effects of recombinant human C1-INH and plasma-derived C1-INH on PBMCs activated by hGIIA

**Fig. 2:**
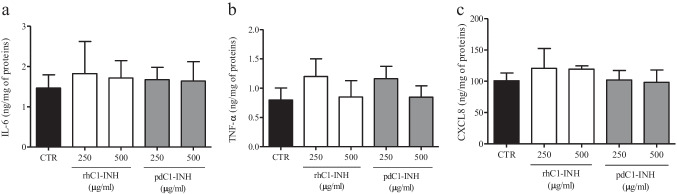
Effects of rhC1-INH and pdC1-INH on secretion of IL-6, TNF-α, and CXCL8 from human PBMCs. PBMCs were stimulated (16 h, 37 °C) with or without the indicated concentrations of rhC1-INH or pdC1-INH. IL-6 (**a**), TNF-α (**b**), and CXCL8 (**c**) concentrations in cell medium were determined by ELISA and values were normalized relative to the amount of total proteins in each well (measured by Bradford assay on cell lysates). The data are reported as mean ± SEM of six different preparations of PBMCs from six different donors

We tested the effects of physiological concentrations of C1-INH on the cytokine/chemokine release from PBMCs. Recombinant human C1-INH (rhC1-INH) and plasma-derived C1-INH (pdC1-INH) had no effect by themselves on the release of IL-6, TNF-α, and CXCL8 (Fig. 2). The presence or absence of 5% FCS in complete medium did not change the effect of C1-INH on cytokine production (data not shown).

**Fig. 3:**
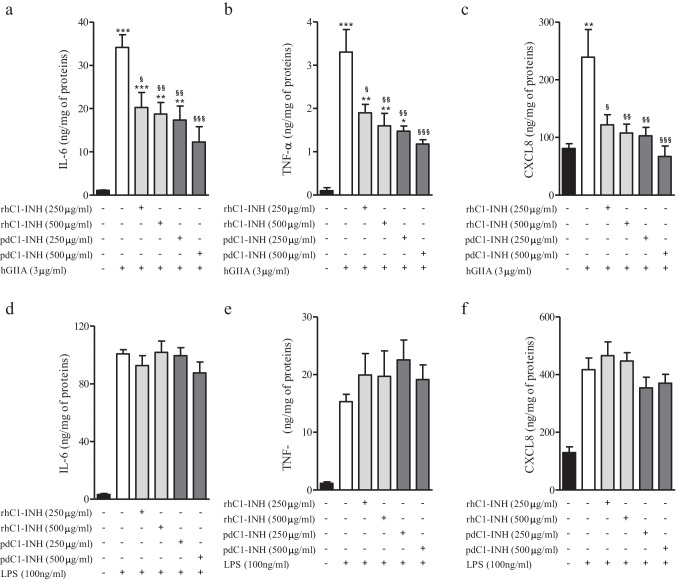
Effect of rhC1-INH and pdC1-INH on hGIIA- or LPS-induced release of IL-6, TNF-α, and CXCL8 from human PBMCs. PBMCs were stimulated (16 h, 37 °C) with hGIIA (**a**–**c**) or LPS (**d**–**f**) alone (white column) or in combination with rhC1-INH (light gray) or pdC1-INH (dark gray). IL-6 (**a**, **d**), TNF-α (**b**, **e**), and CXCL8 (**c**, **f**) release was determined by ELISA and values were normalized relative to the amount of total proteins in each well (measured by Bradford assay on cell lysates). The data are reported as mean ± SEM of six different preparations of PBMCs from six different donors. *p < 0.05 vs. **p < 0.01 and ***p < 0.001 vs. control (black column). ^§^*p* < 0.05 vs. ^§§^*p* < 0.01 and.^§§§^*p* < 0.001 vs. hGIIA, or LPS alone (white column)

However, the same physiological concentrations of rhC1-INH and pdC1-INH dose-dependently inhibited the effect of hGIIA on the release of IL-6, TNF-α, and CXCL8 (Fig. 3a–c). Conversely, rhC1-INH and pdC1-INH had no inhibitory effect on LPS at inducing the secretion of cytokines/chemokines in PBMCs (Fig. 3d–f). The percentage of viable PBMCs at 16 h after treatment with the different stimuli did not differ from that of untreated cells (data not shown).

### hGIIA partially impairs the activity of C1-INH to inhibit C1-esterase

**Fig. 4:**
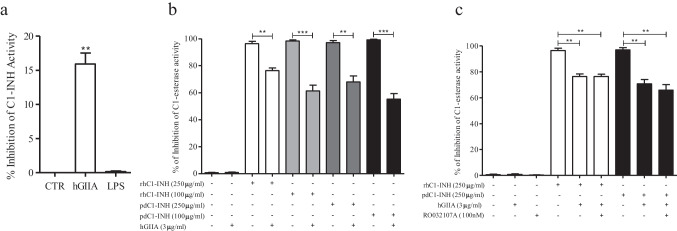
Effects of hGIIA on C1-INH activity. **a** Plasma from normal donors was preincubated (2 h, 37 °C) with or without hGIIA (3 µg/mL) or LPS (100 ng/mL) and the functional activity of C1-INH was then evaluated by a colorimetric assay. Data are expressed as percent inhibition of the maximum plasma activity of C1-INH calculated as (*R* − *R*_*b*_) × 100, where *R* is the C1-INH activity in plasma samples treated with the hGIIA or LPS, and *R*_*b*_ is the C1-INH activity in unstimulated samples. **p < 0.01 vs. control. **b** rhC1-INH and pdC1-INH were incubated (2 h, 37 °C) with or without hGIIA and then functional activity of C1-esterase was evaluated by colorimetric assay. **p < 0.01 vs. rhC1-INH or pdC1-INH alone. **c** hGIIA was preincubated with RO032107A and then incubated with or without rhC1-INH or pdC1-INH (2 h, 37 °C) after which the functional activity of C1-esterase was evaluated. Data are the mean ± SD of 3 experiments. **p < 0.01 vs. rhC1-INH or pdC1-INH alone

Preincubation of plasma from healthy donors (containing C1-INH with normal activity) with hGIIA partially induced an inhibition of C1-INH activity whereas LPS had no effect (Fig. 4a). In another series of experiments, we preincubated rhC1-INH and pdC1-INH with or without hGIIA and measured C1-INH activity. Figure 4b shows that hGIIA alone had no effect on C1-esterase activity but partially reduced the ability of C1-INH molecules to inhibit C1-esterase activity. Moreover, the hGIIA effect was not affected by preincubation of hGIIA with RO032107A (Fig. 4c).

### *Effects of C1-INH on enzymatic activity of sPLA*_*2*_

**Fig. 5:**
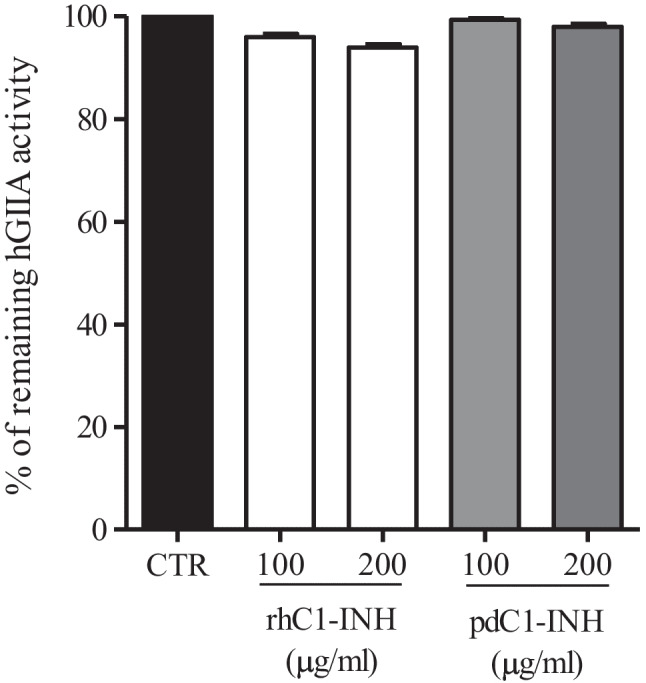
Effect of rhC1-INH and pdC1-INH on hGIIA enzymatic activity. hGIIA (10 pM) was preincubated with the indicated concentrations of recombinant and plasma-derived C1-INH in 100 µL of sPLA_2_ activity buffer for 15 min at room temperature. sPLA_2_ enzymatic activity was measured as reported in the “Materials and methods” section

To identify a possible direct interaction between hGIIA and C1-INH, we tested whether C1-INH modulates the enzymatic activity of hGIIA. When using the highly sensitive radiolabeled *E. coli* membranes sPLA_2_ assay that requires very low concentrations of hGIIA to measure enzymatic activity, rhC1-INH and pdC1-INH had no significant effect on hGIIA enzymatic activity, even at high concentrations of complement inhibitors (Fig. 5).

### C1-INH-hGIIA interaction

**Fig. 6:**
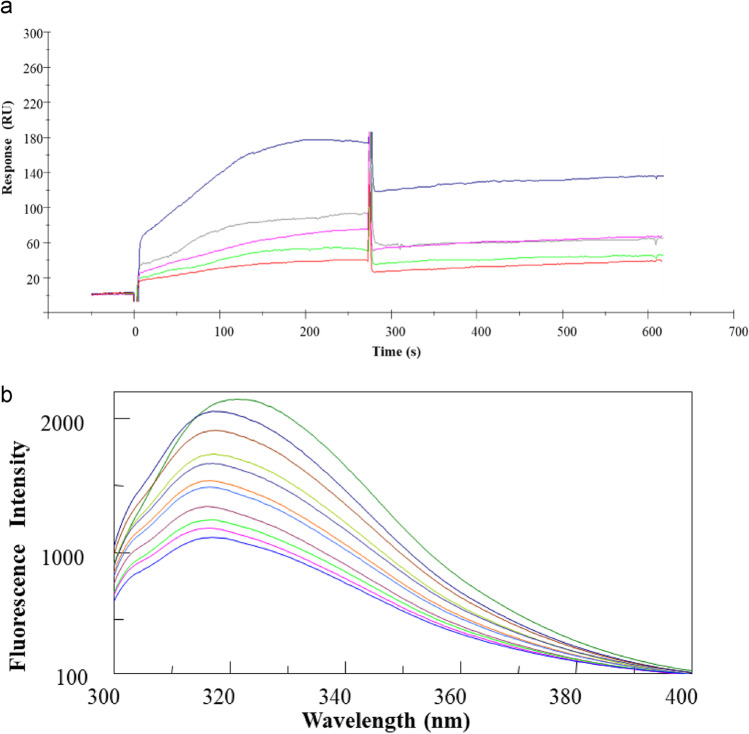
Binding assays for C1-INH-hGIIA interaction: SPR and intrinsic fluorescence. Overlay of **a** sensorgrams recorded at increasing concentrations of C1-INH on hGIIA-chip. **b** Fluorescence emission spectra of C1-INH at increasing equivalents of hGIIA

To further test the possibility of a direct interaction between C1-INH and hGIIA, we first used SPR as in vitro binding assay where hGIIA was immobilized on the sensorchip and pdC1-INH employed as the analyte. The overlay of sensorgrams, reported in Fig. 6a, exhibited a dose–response increase of signal. The kinetic parameters (*k*_on_ = 1.74 × 10^3^ 1/ms and *k*_off_ = 1.25 × 10^−2^ 1/s) allowed to estimate a *K*_*D*_ value of 6.70 µM.

Based on the intrinsic emission of C1-INH and assuming that some aromatic residues could be involved in the formation of the complex C1-INH/hGIIA, we analyzed fluorescence emission spectra of C1-INH at increasing concentrations of hGIIA and the overlay of emission spectra is reported in Fig. 6b. Upon excitation at 298 nm, the emission intensity of C1-INH showed a dose–response quenching and a shift of *λ*_max_ following the addition of hGIIA. This behavior suggested the possible involvement of aromatic solvent-exposed residues in the recognition site between the two proteins, even if specific further studies will be needed to ascertain this finding.

**Fig. 7:**
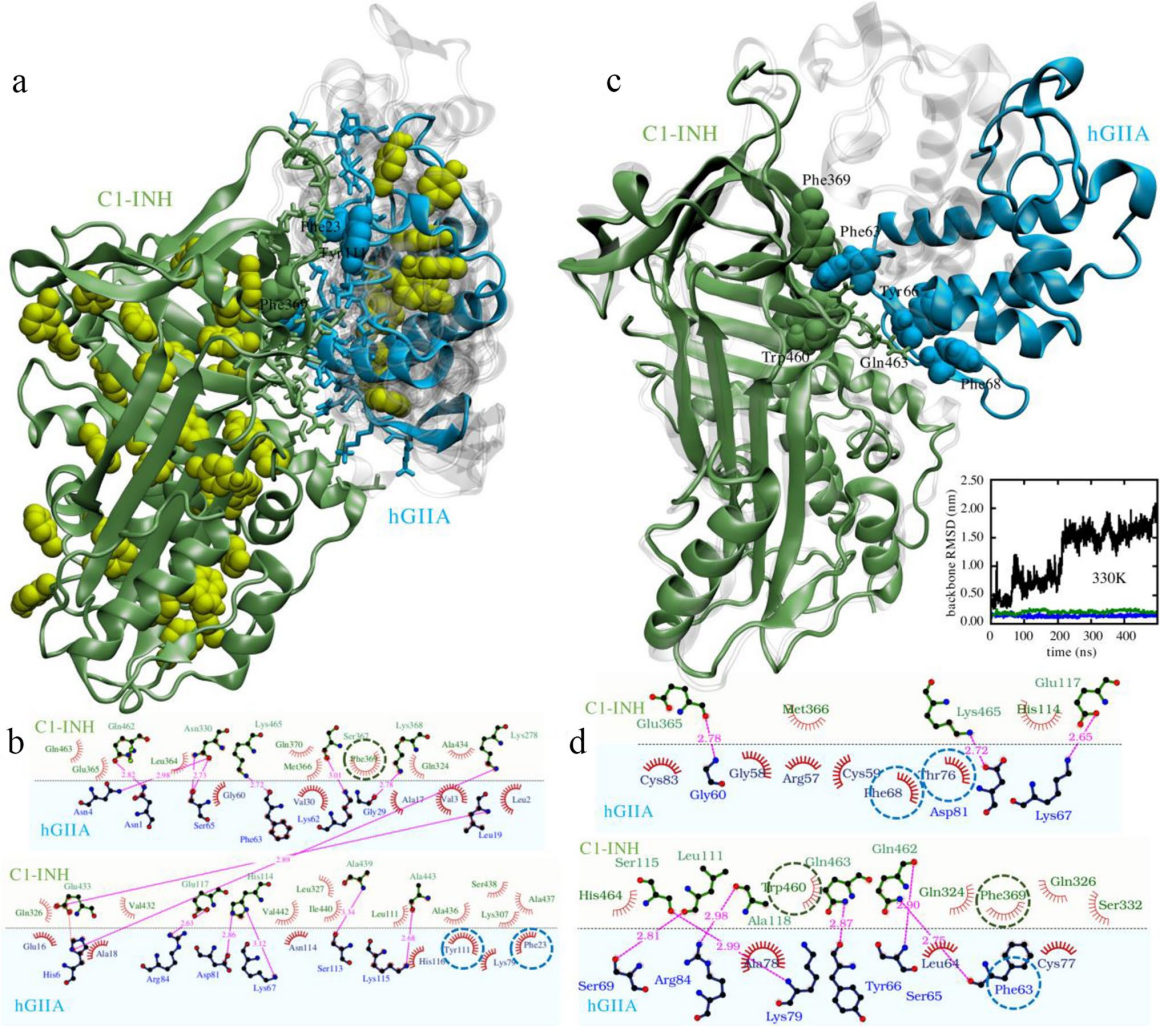
C1-INH:hGIIA complex. **A** Docking poses: predicted conformations of hGIIA (gray shades) on C1-INH (green); the lowest scoring hGIIA conformation is highlighted (cyan). Interacting residues are indicated (licorice) with aromatic side chains further highlighted by their van der Waals spheres. Aromatic residues not involved in the interaction are also indicated (yellow). **B** Schematic diagram of the interaction between C1-INH (top, green shade) and hGIIA (bottom, blue shade) in the optimum HADDOCK pose of panel **A**. **C** Molecular dynamics simulation analysis: comparison between initial (*t* = 0, gray shade) and final (*t* = 500 ns, color) configurations of C1-INH:hGIIA. Simulations were run at *T* = 330 K in full water solvent. Water molecules are not shown for ease of visualization. Interacting aromatic residues are highlighted with their van der Waals spheres. Gln463, forming a hydrogen bond with Tyr66, is also explicitly drawn. In the inset: the backbone RMSD of hGIIA (blue), of C1-INH (green), and of hGIIA with respect to C1-INH (black). **D** Schematic diagram of the interaction between C1-INH (top, green shade) and hGIIA (bottom, blue shade) in final (*t* = 500 ns) configurations of panel **C**. In **A** and **D**, all residues interacting with the target are listed; hydrogen bonds are indicated by dotted lines (magenta). The hydrogen bond distances are also indicated. Aromatic residues are highlighted (dashed circles)

Docking results revealed that 73% of the generated structures (corresponding to the 10 lowest scoring clusters; Fig. 7A) present hGIIA bound to the larger C1-INH binding site comprising Phe-369. Indeed, Phe-369 on C1-INH participates to the binding, as well as Tyr-11 and Phe-23 on hGIIA. Furthermore, in the lowest scoring conformation, the two proteins are kept together by 13 hydrogen bonds (Fig. 7B).

The C1-INH:hGIIA complex stability was then investigated by means of atomistic molecular dynamics simulations in full water solvent. In the simulations, the temperature was kept above room temperature (330 K) to favor molecular rearrangements. The protein complex remained associated along the simulation time and the proteins maintained their conformation as evidenced by their backbone root mean squared deviation (inset in Fig. 7C). However, their reciprocal orientation changed along the simulated time (Fig. 7C). hGIIA moved on C1-INH surface leading to a final observed configuration in which two aromatics are involved on C1-INH: Phe-369 and Trp-460 (Fig. 7D). These form an aromatic interaction with Phe-63 of hGIIA. The involvement of additional hydrogen bonds, such as that between Gln-463 on C1-INH and Tyr-66 on hGIIA, further strengthens the interaction between the two partners. It is important to note that post-translational modifications were not considered in this model. As the N-terminal of C1-INH is believed to serve as an important protein co-factor, the inclusion of the highly glycosylated N-terminal of C1-INH might be a point of interest for further studies to understand its role.

## Discussion

We previously demonstrated the presence of high levels of circulating hGIIA in patients with C1-INH-HAE and a negative correlation between plasma activities of hGIIA and C1-INH [20]. In the present study, we asked whether hGIIA and C1-INH interfere with their respective functions, possibly by a direct protein–protein interaction. First, we found that physiological concentrations of C1-INH inhibit the effect of hGIIA on cytokine and chemokine release from PBMCs. The inhibitory effect of C1-INH was specific and not seen with LPS. Second, we found that hGIIA partially inhibited the function of C1-INH at inhibiting C1-esterase activity, suggesting a direct protein–protein interaction between hGIIA and C1-INH. The results of surface plasmon resonance and spectroscopic experiments as well as in silico docking and molecular dynamics analyses strengthen the possible interaction between the two proteins assuming that some aromatic residues may be involved in the interaction of C1-INH with hGIIA. Interestingly, C1-INH did not inhibit the enzymatic activity of hGIIA, while RO032107A, a potent small molecule inhibitor of hGIIA, had a significant inhibitory effect on hGIIA-induced cytokine release, similar to that of C1-INH. hGIIA reduced the ability of plasma-derived and recombinant C1-INH to inhibit C1-esterase activity but the preincubation of hGIIA with RO032107A did not affect this property. Together, this suggests that C1-INH may interact directly with hGIIA via a binding surface different from the active site or has a more complex effect on hGIIA, both direct and indirect. Further study will be necessary to demonstrate this hypothesis.

Patients with C1-INH-HAE are characterized by functional and/or antigenic C1-INH deficiency. These patients exhibit a wide spectrum of residual functional activity of C1-INH between 0 and 50% of normal C1-INH functional activity of healthy people. We hypothesized that the differences in the complement component level in C1-INH-HAE patients could be caused by the presence of plasma hGIIA in variable amounts [20] that would contribute to inhibition of C1-INH activity [52]. Moreover, we dare the hypothesis that a rapid increase of circulating plasma hGIIA could induce a transient drop of C1-INH activity and contribute to development of angioedema.

In this manuscript, we also show that both rhC1-INH and pdC1-INH inhibit the effect of hGIIA on the production of cytokines from PBMCs at concentrations found in healthy donors. It will be interesting to test whether plasma from C1-INH-HAE patients with various levels of hGIIA versus C1-INH and complement produce various levels of cytokine production by PBMCs, thereby showing a complex interplay between these three factors. Such a scenario would be in line with protein complexes consisting of several proteins and playing important role in regulatory processes, cellular activation, and signaling cascades [52]. It is also in line with the fact that hGIIA can act on cells through either enzymatic activity [10] or its ability to interact with different targets including heparan sulfate proteoglycans (HSPGs) and integrins [5, 9, 14, 16, 17, 19, 53–56]. This study only analyzed the effect of hGIIA on PBMCs while hGIIA and other sPLA_2_s can activate several blood and resident immune cells such as neutrophils, macrophages, eosinophils, and platelets [5, 6, 57–59]. Thus, in the future, it will be interesting to study the effect of C1-INH on the biological roles of hGIIA in different pathophysiological conditions, and to expand our findings to test whether C1-INH impacts on the effect of various sPLA_2_s in the activation of other immune cells. In particular, it is well demonstrated that high levels of circulating sPLA_2_ are found in several pathological situations and positively correlate with disease severity [9, 16, 17, 19]. Interestingly, PLA_2_ serum activity is increased in B-cell lymphoma and has been proposed as a new biomarker for B-cell lymphoproliferation [60]. Moreover, we know that acquired angioedema due to C1-inhibitor deficiency (AAE-C1-INH) is often associated with malignant B-cell lymphoma and other disorders [61]. Therefore, it is conceivable that the increase of circulating PLA2 in lymphoma could be the cause of decrease of C1-INH and so development of AAE-C1-INH. Further studies are needed to demonstrate this hypothesis.

In conclusion, since C1-INH inhibits the proinflammatory effect of sPLA_2_, the data collected in this paper suggest that patients with angioedema with C1-INH deficiency could have a greater and uncontrolled inflammatory response to endogenous (human) and exogenous (bee and snake venom, etc.) sPLA_2_s compared to healthy subjects. Further study will be necessary to demonstrate this hypothesis.

## Data Availability

Data and material supporting the reported results are available upon request.

## Abbreviations

FXIIa: Activated factor XII
FXIa: Activated FXI
PKa: Activated plasma kallikrein
*k*_on_: Association rate value
C1-INH: C1-esterase inhibitor
CD: Circular dichroism
CXCL8: C-X-C motif chemokine ligand 8
*k*_off_: Dissociation rate value
FCS: Fetal calf serum
HAE: Hereditary angioedema
pdC1-INH: Human plasma–derived C1-INH
hGIIA: Human group IIA sPLA_2_
IL-6: Interleukin-6
LPS: Lipopolysaccharide
MASP-1: MBL-associated serine protease 1
PBMCs: Peripheral blood mononuclear cells
rhC1-INH: Recombinant human C1-INH
sPLA_2_: Secreted phospholipase A_2_
TNF-α: Tumor necrosis factor-alpha
